# The application of artificial intelligence in diagnosis of Alzheimer’s disease: a bibliometric analysis

**DOI:** 10.3389/fneur.2024.1510729

**Published:** 2024-12-05

**Authors:** Xiaoqiong An, Jun He, Bin Bi, Gang Wu, Jianwei Xu, Wenfeng Yu, Zhenkui Ren

**Affiliations:** ^1^Department of Laboratory Medicine, The Second People's Hospital of Guizhou Province, Guiyang, China; ^2^Guizhou Provincial Center for Clinical Laboratory, Guiyang, China; ^3^Psychosomatic Department, The Second People's Hospital of Guizhou Province, Guiyang, China; ^4^Center for Tissue Engineering and Stem Cell Research, Guizhou Medical University, Guiyang, China; ^5^Department of Pharmacology, School of Basic Medicine, Guizhou Medical University, Guiyang, China; ^6^Key Laboratory of Molecular Biology, Guizhou Medical University, Guiyang, China; ^7^Key Laboratory of Human Brain Bank for Functions and Diseases of Department of Education of Guizhou Province, Guizhou Medical University, Guiyang, China; ^8^Laboratory Department of People’s Hospital of Southwest Guizhou Autonomous Prefecture, Xingyi, China

**Keywords:** Alzheimer’s disease, artificial intelligence, Bibliometrix, CiteSpace, VOSviewer

## Abstract

Alzheimer’s disease (AD) is a neurodegenerative disorder that severely impacts cognitive function, posing significant physical and psychological burdens on patients and substantial economic challenges to families and society, particularly in aging populations where its prevalence is rising. Current diagnostic and therapeutic strategies, including pharmacological treatments and non-pharmacological interventions, exhibit considerable limitations in early diagnosis, etiological treatment, and disease management. This study aims to investigate the application of artificial intelligence (AI) in the early diagnosis and progression monitoring of AD through a bibliometric analysis of relevant literature. A systematic search in the Web of Science Core Collection identified 530 publications related to AI and AD, consisting of 361 original research articles and 169 review articles, with a notable increase in annual publication rates, particularly between 2019 and 2024. The United States and China emerged as leading contributors, emphasizing the importance of international collaboration. Institutional analysis revealed that Harvard University and Indiana University System are at the forefront, highlighting the role of academic institutions in fostering interdisciplinary research. Furthermore, the Journal of Alzheimer’s Disease was identified as the most influential publication outlet. Key highly cited papers provided essential theoretical foundations for ongoing research. This study underscores the growing relevance of AI in AD research and suggests promising avenues for future investigations, particularly in enhancing diagnostic accuracy and therapeutic strategies through advanced data analytics and machine learning techniques.

## 1 Introduction

Alzheimer’s disease (AD) is a progressive neurodegenerative disorder characterized by cognitive decline, memory loss, and behavioral changes, significantly impacting patients and their families, as well as imposing a substantial economic burden on healthcare systems worldwide (1, 2). The increasing prevalence of AD, projected to affect over 150 million individuals by 2050, necessitates urgent advancements in diagnostic and therapeutic strategies (3, 4). Current approaches primarily rely on clinical assessments and neuroimaging techniques, which, while valuable, often fall short in early detection and personalized treatment, leading to delayed interventions and suboptimal patient outcomes.

Despite substantial investments in drug development aimed at AD, there has been a conspicuous lack of breakthrough advancements, with existing treatments often yielding suboptimal outcomes (5). This stagnation underscores the urgent need for innovative approaches to enhance our understanding of AD’s complex pathophysiology and to identify potential therapeutic targets (6). Previous studies have elucidated various mechanisms underlying AD, including amyloid-beta accumulation, tau protein hyperphosphorylation, and neuroinflammation, yet these findings have not translated into effective clinical solutions (7, 8, 9).

The integration of artificial intelligence (AI) into the diagnosis and treatment of Alzheimer’s disease has emerged as a pivotal advancement in the field of neurology and geriatric medicine (10, 11). By analyzing vast datasets derived from neuroimaging, genetic information, and clinical assessments, AI systems can identify biomarkers that may be overlooked by traditional diagnostic methods (12). Furthermore, AI-driven tools facilitate the development of predictive models that can forecast the onset of Alzheimer’s disease in at-risk populations, thereby enabling early intervention and improved patient outcomes (13). The application of AI in this context not only streamlines clinical workflows but also fosters a more nuanced understanding of the complex pathophysiology of Alzheimer’s disease, ultimately contributing to the advancement of precision medicine in neurodegenerative disorders.

## 2 Materials and methods

### 2.1 Data source and search strategy

**Figure 1:**
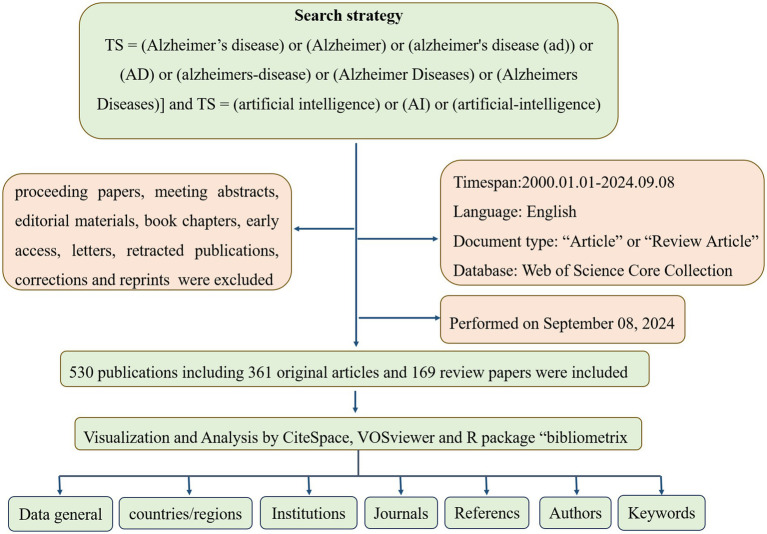
Flowchart of the bibliometric search and analysis process.

Due to its comprehensive coverage of over 12,000 academic journals and its frequent use by researchers, Web of Science was selected as the primary database for this study. Compared with other databases such as Scopus, Medline, and PubMed, Web of Science provides the most comprehensive and reliable bibliometric analysis (14). The Web of Science Core Collections Database (WOSCC) was searched on September 8, 2024, for literature on artificial intelligence and Alzheimer’s disease published between January 1, 2010, and September 8, 2024. To reduce the bias caused by frequent database updates, we completed the search in 1 day. There was a search formula set as follows: [TS = (Alzheimer’s disease) or (Alzheimer) or (Alzheimer’s disease (AD)) or (AD) or (Alzheimer’s disease) or (Alzheimer Diseases) or (Alzheimer’s Diseases)] and [TS = (artificial intelligence) or (AI) or (artificial-intelligence)] and [LA = (English)]. In this study, only articles and reviews were considered (editorials, proceedings papers, abstracts, and book chapters were excluded), with a time frame from January 1, 2010, to September 8, 2024. Figure 1 illustrates how paper screening is conducted. Finally, in order to ensure data quality, we perform data cleansing, which includes the removal of duplicate records, uniform formatting of author names and dates, and handling of missing values. For records with critical information missing, we adopted a strategy of either filling in or excluding the records.

### 2.2 Statistical analysis

Bibliometric analysis often involves the application of mathematical and statistical methods to scrutinize research outcomes, with the goal of extracting meaningful information and identifying dominant patterns within extensive scholarly works (15, 16). This analytical approach offers insights into various bibliometric dimensions, including countries, institutions, journals, authors, keywords, and citations, presenting a holistic dataset encompassing these elements (17). Moreover, through visualization, it becomes possible to evaluate the ongoing research advancements in specific domains and to foresee emerging trends and focal points (18). In the field of bibliometrics, VOSviewer, CiteSpace, and Bibliometrix are three software tools designed for the analysis of scientific literature networks (19).

VOSviewer is a software tool for constructing and visualizing scientific literature networks (20). It processes citation, co-citation, co-word, and co-authorship data, generating intuitive scientific maps that help researchers identify research trends and knowledge structures (21). The software offers three types of visualizations: network view, overlay view, and density view (22). In our study, we conducted an in-depth bibliometric analysis using VOSviewer software. Initially, we set the analysis parameters based on our research objectives, including the time frame, node types, relationship strengths, threshold, clustering algorithm, clustering parameters, and visualization parameters to ensure the precision and relevance of the results. Subsequently, the software automatically constructed collaboration and co-citation networks based on these parameters, visually displaying the interrelationships and knowledge flow among literature. Finally, we generated various charts, including collaboration and co-citation network diagrams, which revealed the structure and dynamics within the research field. Through this process, we were able to clearly present and analyze key collaborations and knowledge development paths within the research area.

CiteSpace, based on the Java platform, it offers an intuitive approach to analyzing and visualizing large volumes of literature data, especially suitable for handling information from various academic database and helps researchers discover research hotspots and future trends in scientific literature (23, 24). In this study, we employed CiteSpace version 6.3.R1 to perform a comprehensive bibliometric analysis, which included the generation of clustering maps to visualize reference and keyword relationships and timeline visualizations to track the evolution of references over time. The software also enabled us to identify citation bursts within references, indicating significant research waves. Additionally, we conducted a dual-map analysis of journals to explore shifts in research directions and trends, revealing key themes and influential journals in the field. To achieve this, we set parameters such as time span, node types, relationship strengths, and co-occurrence thresholds, and leveraged CiteSpace’s clustering algorithms, including LSI, LLR, and MI, to identify research topics and trends. The software facilitated the extraction of terms from the titles, keywords, and abstracts of the literature, allowing us to construct collaboration and co-citation networks. These networks were then used to generate intuitive charts that illuminated the structure and dynamics of the research field, providing a clear overview of the key topics and influential publications.

Bibliometrix is an R package that covers the entire workflow of scientific mapping, from data collection to analysis and visualization (25). These tools enable researchers to identify research hotspots, trends, and patterns, providing valuable insights for academic studies. In addition, a bibliometric platform^1^ is used to visualize international collaboration.

## 3 Results

### 3.1 Data general

**Figure 2:**
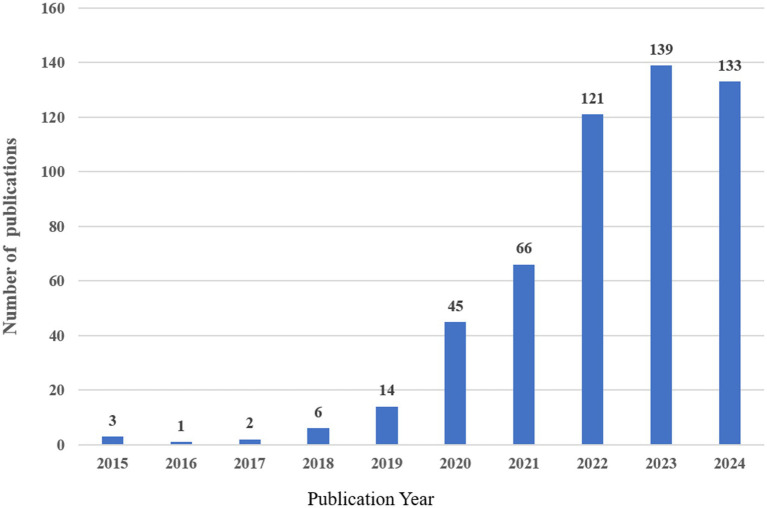
Annual worldwide publication output.

Following our search criteria, we identified a total of 530 research works related to artificial intelligence and Alzheimer’s disease since 2010, comprising 361 original articles and 169 review papers. There has been a consistent annual increase in the number of publications, with a particularly dramatic surge observed between 2019 and 2024. Even with only partial data for 2024, the count has already exceeded 133 articles, indicating a persistent upward trajectory. However, there was a negligible amount of related research prior to 2015 (Figure 2).

### 3.2 An analysis of the most productive countries/regions

**Figure 3:**
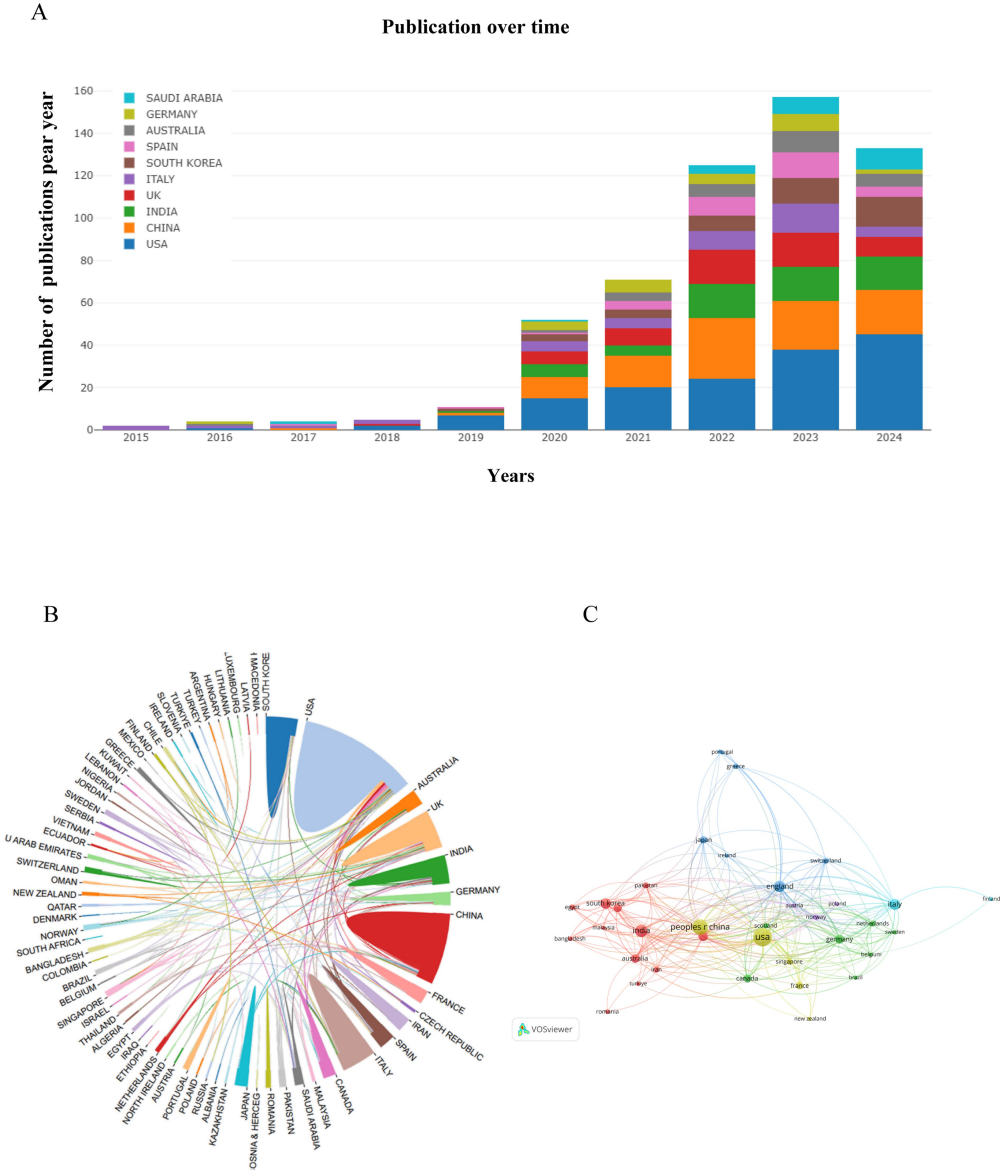
Visualization of countries/regions in the research of AI-related AD during 2010–2024. **(A)** The annual number of publications in major countries. **(B)** Publication distribution and collaboration between countries/regions. **(C)** Country/regional collaboration network. In the country/regional collaboration network, nodes represent individual countries or regions, and their sizes correspond to the publication volumes of each entity. The nodes of different colors represent the countries/regions with different clusters.

A total of 76 countries/regions participated in research on artificial intelligence and Alzheimer’s disease between 2010 and 2024. The top 10 countries/regions with significant contributions and notable associations are presented in Table 1, it can reflect the countries/regions that have made the most significant contributions in this area. Figure 3A and Table 1 show the top 10 countries and institutions according to publication number, and the top 5 countries are the United States, China, India, England and Italy. Table 1 indicates that the United States has a leading role with 152 publications, highlighting its significant contribution to the field. China follows with a notable 100 publications, indicating a strong research presence. India’s 60 publications reflect an emerging research force, while England’s 50 and Italy’s 44 demonstrate their active engagement in the area. In the analysis of citations per publication, Germany secured the top position with an average of 20.88 citations per publication, followed closely by Italy with 18.18 citations per publication. The United States and England made significant advancements, claiming the third and fourth spots with 17.86 and 17.04 citations per publication, respectively. Additionally, we examined the collaborative ties between nations, with lines connecting two countries signifying the presence of cooperative relationships, the analysis of the global country collaboration network illustrates the interconnectedness and collaborative efforts among various nations in the field. As illustrated in Figures 3B,C, the analysis revealed a pattern of intensified collaboration among a select group of countries. Specifically, the United States, the United Kingdom, Canada, India, China, and France exhibited a higher degree of cooperative engagement with one another. The strength of these connections implies a significant impact on the collective advancement of research and innovation in their respective fields of study.

**Table 1:** Top 10 countries/regions publishing research related to AI in Alzheimer’s disease.

Rank	Country/region	Publications	Citations	citations per publication	Total link strength	Links
1	United States	152	2,714	17.86	121	32
2	Peoples R China	100	1,253	12.53	64	23
3	India	60	628	10.47	54	21
4	England	50	852	17.04	93	27
5	Italy	44	800	18.18	52	22
6	South Korea	41	422	10.29	30	14
7	Spain	33	510	15.45	55	26
8	Australia	28	392	14.00	38	20
9	Germany	26	543	20.88	51	20
10	Saudi Arabia	24	188	7.83	33	16

### 3.3 An analysis of the most productive institutions

**Figure 4:**
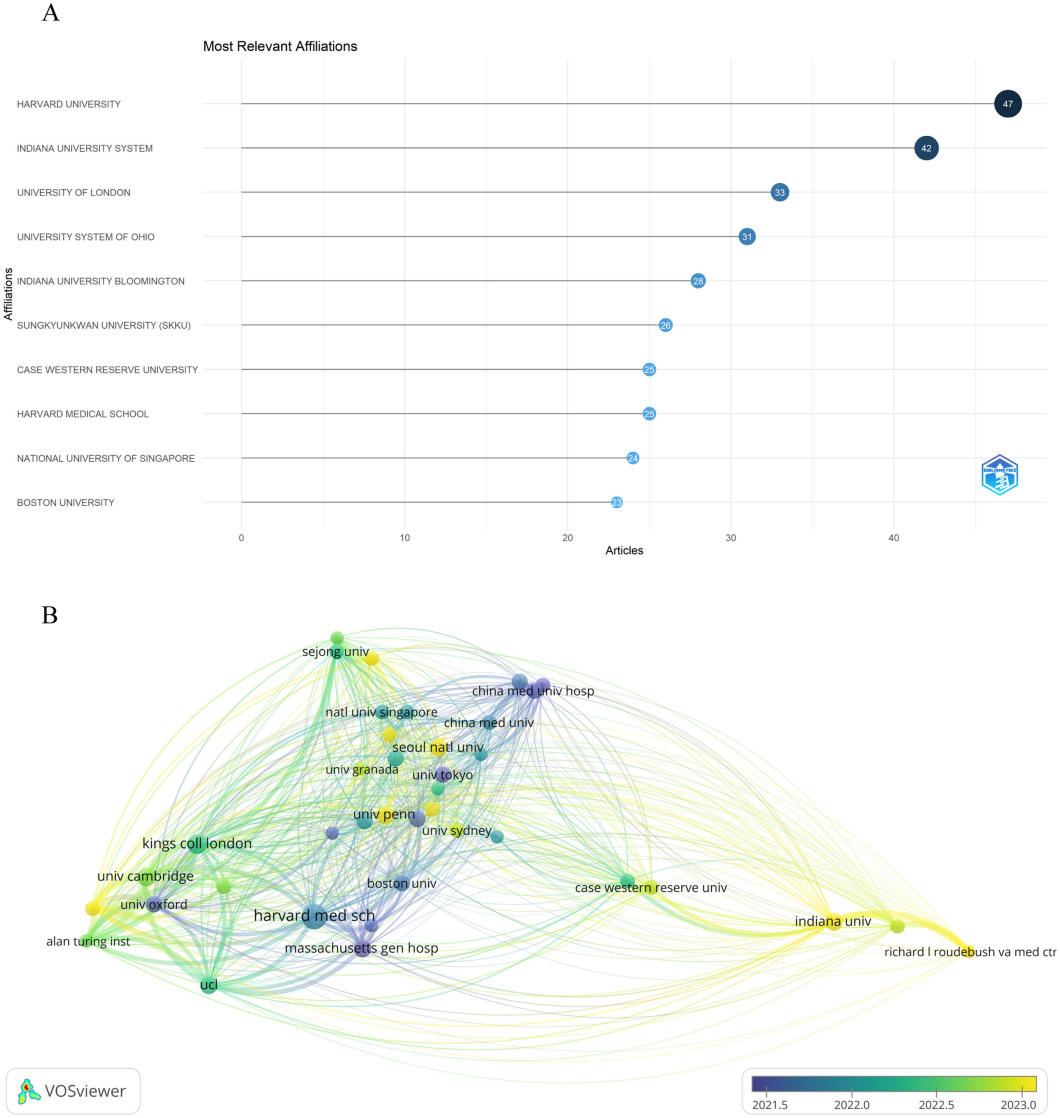
Analysis of institutions engaged in research on AI-related AD. **(A)** Top 10 most productive institutions based on publication performance. **(B)** Overlay visualization map of bibliographic coupling analysis of institutions. The size of each institution’s name in the network diagram is proportional to the number of articles published by that institution. The curvature and thickness of the lines connecting institutions indicate the strength of their collaborative ties. Additionally, the circle’s color gradients represent different time intervals within the study period.

It is estimated that over 1,228 institutions have contributed to the research on artificial intelligence and Alzheimer’s disease. As shown in Figure 4A, seven of the top 10 most productive institutions were from the United States, as Harvard University ranks first with 47 publications, followed by Indiana University System in second position with 42 articles. In addition, the institutions with the third highest number of publications are University of London from the United Kingdom, with 33 articles published. It is clear that both the United States and the United Kingdom have significant influence in this field. In addition, we performed a bibliographic coupling analysis to explore the intellectual and research connections among institutions with a minimum threshold of 5 publications, as depicted in Figure 4B, from the 1,228 institutions initially considered, 39 met the criteria for inclusion in the analysis. This method, which discerns relationships through the co-citation of documents, offers a graphical depiction of the inter-institutional scholarly links. Harvard Medical School emerged with the most substantial total link strength, totaling 4,319, succeeded by University of Cambridge with 4,235, and the Alan Turing Institute with 3,880. Additionally, the overlay map visualization indicates that research on artificial intelligence and Alzheimer’s disease has only rapidly developed in recent years, demonstrating the immense potential of artificial intelligence technology in the early diagnosis of Alzheimer’s disease, monitoring disease progression, developing treatment strategies, and researching pathological mechanisms in the future.

### 3.4 An analysis of the journals and co-cited journals with the highest impact

**Figure 5:**
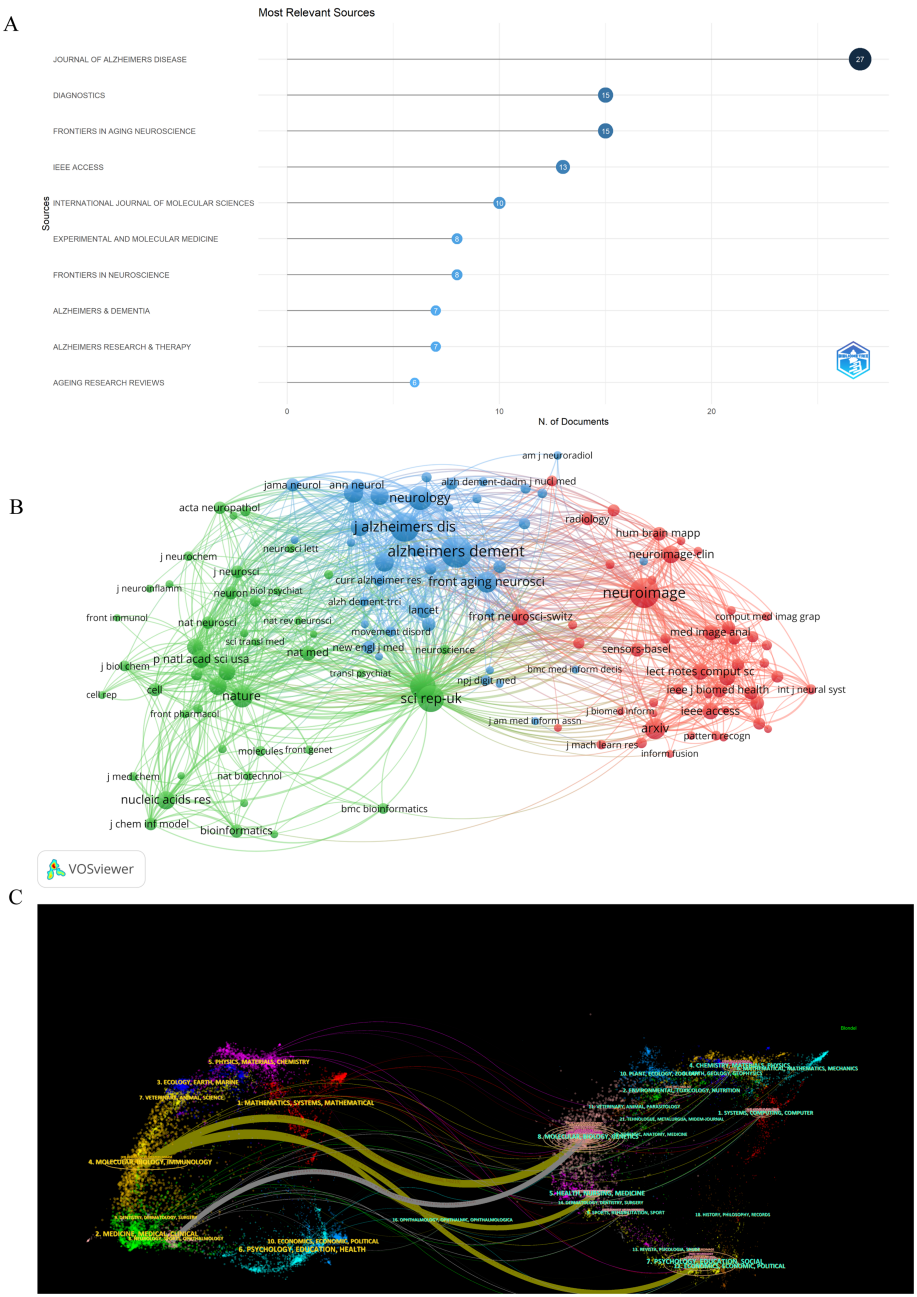
Analysis of journals and co-cited-journals engaged in research on AI-related AD. **(A)** The number of publications of the top 10 journals. **(B)** Co-cited journal analysis displays the relationships between journals with a co-citation count of 50 or more. The size of each journal node corresponds to the number of articles published by that journal. The thickness of the curved lines connecting the nodes represents the level of collaboration between the journals. The color coding within the nodes indicates different clusters, each representing a group of journals with similar research affinities. **(C)** Double graphical overlay of journals. On the left were the citing journals, on the right were the cited journals, and the colored path represented the citation relationship.

A total of 282 distinct journals have published 530 articles in the field of artificial intelligence and Alzheimer’s disease. Among these journals, five have over 10 papers published. Table 2 shows the top 10 journals/co-cited journals with the number of publications and citations/co-citations, and their corresponding Impact Factor (JCR 2023) and JCR quartile. As depicted in Figure 5A and Table 2, the top 10 journals with the highest number of published articles are showcased. This ranking reflects the prolific contribution of these journals to the field, indicating their significant role in disseminating research findings and advancing scientific knowledge. Notably, the Journal of Alzheimer’s Disease (IF = 3.4, Q2) stands out prominently in the field, contributing 27 publications and a substantial total link strength of 346, emphasizing its significant role in advancing research on Alzheimer’s disease. Closely trailing, the Diagnostics (IF = 3, Q1) exhibits a notable presence as well, publishing 15 articles and achieving a total link strength of 154. Furthermore, the Frontiers in Aging Neuroscience (IF = 4.1, Q2) has made a remarkable impact, published 15 articles, demonstrating an even higher total link strength of 434. These journals, frequently cited in the realm of Alzheimer’s disease research, underscore their influential positions not only in the broader neuroscience community but also within the intersection of artificial intelligence and Alzheimer’s disease studies.

**Table 2:** The top 10 journals and the co-cited journals that published documents on AI related to AD.

Rank	Sources	Articles	Citations	Total link strength	IF (2023)	JCR (2023)	Co-cited journal	Co-citations	Total link strength	IF (2023)	JCR (2023)
1	Journal of Alzheimers Disease	27	346	21	3.2	Q2	Alzheimers Dement	820	11,559	13	Q1
2	Diagnostics	15	154	17	3	Q1	Neuroimage	740	9,194	4.7	Q1
3	Frontiers in Aging Neuroscience	15	434	22	4.1	Q2	J Alzheimers Dis	696	11,031	3.4	Q2
4	IEEE Access	13	177	5	3.4	Q2	Sci Rep-UK	597	7,755	3.8	Q1
5	International Journal of Molecular Sciences	10	164	8	4.9	Q2	Plos One	542	7,975	2.9	Q1
6	Experimental and Molecular Medicine	8	23	0	9.5	Q2	Neurology	463	7,282	7.7	Q1
7	Frontiers in Neuroscience	8	338	12	3.2	Q1	Nature	380	4,564	50.5	Q1
8	Alzheimers and Dementia	7	116	10	13	Q1	Front Aging Neurosci	363	5,332	4.1	Q2
9	Alzheimers Research and Therapy	7	122	5	7.9	Q2	Arxiv	362	3,037		
10	Ageing Research Reviews	6	49	7	12.5	Q1	Neurobiol Aging	318	6,721	3.7	Q2

Journals that have garnered citations from multiple authors are designated as co-cited journals. As depicted in Figure 5B, we conducted a visual analysis focusing on these co-cited journals that received over 50 citations. Among them, the Alzheimer’s & Dementia emerged as the top contender, boasting an Impact Factor of 13 and a remarkable 820 citations. This was closely followed by the Neuroimage, with an Impact Factor of 4.7 and 740 citations, and the Journal of Alzheimer’s Disease, holding an Impact Factor of 3.4 and 696 citations. These journals not only demonstrate their widespread influence within the field but also underscore their importance as key sources of information and research insights. Table 2 provides detailed information on journals and co-cited journals, which allows you to explore publication preferences among authors and provide useful references for future research.

Using the double-map overlay of journals shown in Figure 5C, we can see the left side displays the distribution of journals publishing the citing literature, reflecting the primary disciplines of the cited articles. The right side shows the journals where the cited literature appears, indicating the disciplines that frequently reference the material. Colored lines denote the citation connections between them.

### 3.5 An analysis of the cited references and co-cited references

In order to build on existing knowledge and concepts, researchers need to know which works have gained the most attention and citations. Table 3 presents the top 10 most cited articles in the field of artificial intelligence and Alzheimer’s disease research, with citation counts ranging from 103 to 321. The document with the highest global citations, “Alzheimer’s disease drug development pipeline: 2022” authored by Jeffrey Cummings and colleagues, appeared in the journal Alzheimers Dement (N Y) in 2022 (26). Following in second place is “Deep Learning in Alzheimer’s Disease: Diagnostic Classification and Prognostic Prediction Using Neuroimaging Data” by Taeho Jo and team, which was published in Frontiers in Aging Neuroscience in 2019 (27), garnering 281 citations. In third position, “Recent Advancements in Pathogenesis, Diagnostics and Treatment of Alzheimer’s Disease” by Sahil Khan and co-authors, earned 272 citations upon its publication in Current Neuropharmacology in 2020 (1).

**Table 3:** The top 10 most globally cited documents.

Rank	Literature	Total citations	Author	Year	Journal	IF (2023)	JCR (2023)	DOI
1	Alzheimer’s disease drug development pipeline: 2022	321	Jeffrey Cummings	2022	Alzheimers Dement (N Y)	Q1	4.9	10.1002/trc2.12295
2	Deep Learning in Alzheimer’s Disease: Diagnostic Classification and Prognostic Prediction Using Neuroimaging Data	281	Taeho Jo	2019	Front Aging Neurosci	Q2	4.1	10.3389/fnagi.2019.00220
3	Recent Advancements in Pathogenesis, Diagnostics and Treatment of Alzheimer’s Disease	272	Sahil Khan	2020	Curr Neuropharmacol	Q1	4.8	10.2174/1570159X18666200528142429
4	COVID-19 Artificial Intelligence Diagnosis Using Only Cough Recordings	252	Jordi Laguarta	2020	IEEE Open J Eng Med Biol	Q3	2.7	10.1109/OJEMB.2020.3026928
5	Global Evolution of Research in Artificial Intelligence in Health and Medicine: A Bibliometric Study	190	Bach Xuan Tran	2019	J Clin Med	Q1	3.0	10.3390/jcm8030360
6	Magnetic resonance imaging biomarkers for the early diagnosis of Alzheimer’s disease: a machine learning approach	156	Christian Salvatore	2015	Front Neurosci	Q2	3.2	10.3389/fnins.2015.00307
7	Diagnosis of Alzheimer’s Disease via Multi-Modality 3D Convolutional Neural Network	130	Yechong Huang	2019	Front Neurosci	Q2	3.2	10.3389/fnins.2019.00509
8	A multilayer multimodal detection and prediction model based on explainable artificial intelligence for Alzheimer’s disease	124	Shaker El-Sappagh	2021	Scientific Reports	Q1	3.8	10.1038/s41598-021-82098-3
9	Neuroimaging advances regarding subjective cognitive decline in preclinical Alzheimer’s disease	115	Xiaoqi Wang	2020	Mol Neurodegener	Q1	14.9	10.1186/s13024-020-00395-3
10	Machine Learning-based Virtual Screening and Its Applications to Alzheimer’s Drug Discovery: A Review	103	Kristy A Carpenter	2018	Curr Pharm Des	Q2	2.6	10.2174/1381612824666180607124038

**Figure 6:**
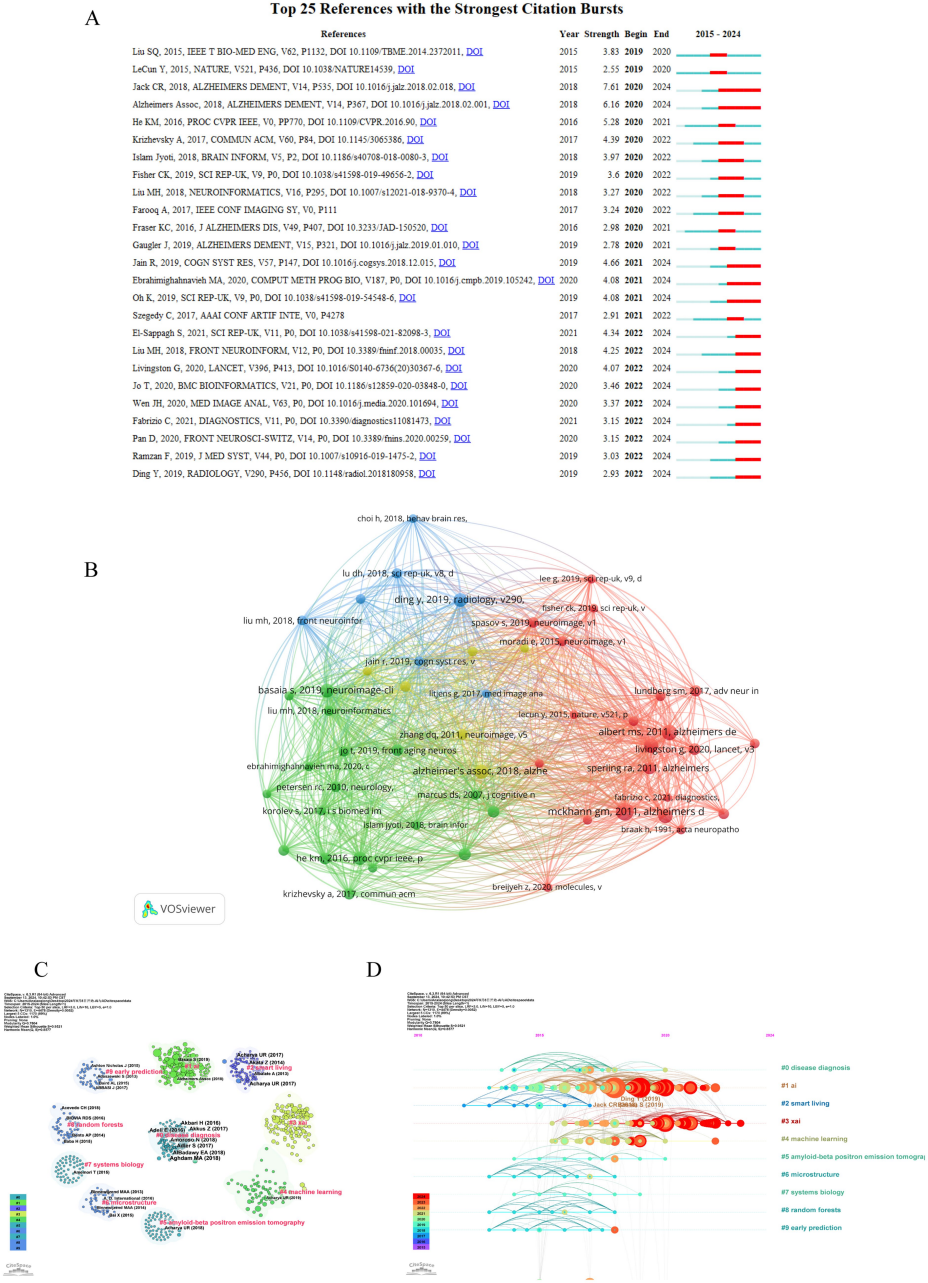
Visualization analysis of cited and co-cited references. **(A)** Cluster analysis of co-cited references. Different colors represent different clusters of co-cited references. **(B)** Representative burst citations in the top 25 references with the most powerful citation burst. **(C)** Co-cited references were grouped into clusters with dots denoting references and different colors denoting different clusters. **(D)** Timeline distribution of the top10 clusters.

A co-citation network is a graphical depiction of how frequently documents are cited together, indicating a conceptual link between them. Conceptual clusters form within this network when certain documents are consistently co-cited, signifying a shared research theme or focus. These clusters help identify central topics and influential works within an academic field. The top 10 co-cited references in the artificial intelligence and Alzheimer’s disease field are listed in Table 4. The most co-cited article is “The diagnosis of dementia due to Alzheimer’s disease: recommendations from the National Institute on Aging-Alzheimer’s Association workgroups on diagnostic guidelines for Alzheimer’s disease (28)” (55 citations), followed by “The diagnosis of mild cognitive impairment due to Alzheimer’s disease: recommendations from the National Institute on Aging-Alzheimer’s Association workgroups on diagnostic guidelines for Alzheimer’s disease (29)” (42 citations). The third most co-cited article is “Mini-mental state. A practical method for grading the cognitive state of patients for the clinician” and “NIA-AA Research Framework: Toward a biological definition of Alzheimer’s disease (30)” (38 citations). In order to construct a map of co-citations, references with a co-citation count of at least 15 were selected, a total of 52 references were selected for analysis of co-citations (Figure 6A). There were different colors for different clusters of references. The first cluster (in red) included 21 references, while the second cluster (in green) included 18 references. Cluster 3 (in blue) included 7 references and Cluster 4 (in yellow) included 6 references. Citation surge denotes a rapid escalation in the frequency of citations within a brief timeframe, indicating the focal points of research interest for a specific duration and tracking the evolution of these focal points across different time periods. This phenomenon is also utilized to assess the dynamics and trajectory of emerging research trends. The top 25 references with the strongest citation burst are shown in Figure 6B. Among these, the publication titled “NIA-AA Research Framework: Toward a biological definition of Alzheimer’s disease (30)” authored by Clifford R Jack Jr. and colleagues in 2018 in the journal Alzheimers Dement, exhibited the most pronounced citation burst (strength = 7.61), with the surge occurring between 2020 and 2024. This review proposes a biomarker-based definition of Alzheimer’s disease, viewing it as a continuous pathological process and providing researchers with a common language to explore the intreractions between pathological changes and cognitive symptoms. In addition, we utilized CiteSpace to visualize the co-citation network of references, spanning the years 2010 to 2024 with yearly intervals, highlighting the key reference nodes. As shown in Figure 6C, each dot represents a reference, and they were grouped into different clusters based on their intersecting research areas, “disease diagnosis” (#0) is the largest cluster, followed by “AI” (#1), “smart living” (#2) and “xai” (#3). Subsequently, the timeline of key clusters was charted using CiteSpace, as displayed in Figure 6D, a temporal analysis shows that the hotspots of research have shifted from the original #9 early prediction, #8 random forests, #6 microstructure and #2 smart living to #1 ai, #3 xai, #4 machine learning, and #0 disease diagnosis.

**Table 4:** The top 10 most globally cited references.

Rank	Cited references	Co-cited counts	Author	Year	Journal	IF (2023)	JCR (2023)	DOI
1	The diagnosis of dementia due to Alzheimer’s disease: recommendations from the National Institute on Aging-Alzheimer’s Association workgroups on diagnostic guidelines for Alzheimer’s disease	55	Guy M McKhann	2011	Alzheimers Dement	Q1	13.1	10.1016/J.JALZ.2011.03.005
2	The diagnosis of mild cognitive impairment due to Alzheimer’s disease: recommendations from the National Institute on Aging-Alzheimer’s Association workgroups on diagnostic guidelines for Alzheimer’s disease	42	Marilyn S Albert	2011	Alzheimers Dement	Q1	13.1	10.1016/J.JALZ.2011.03.008
3	“Mini-mental state”. A practical method for grading the cognitive state of patients for the clinician	38	M F Folstein	1975	J Psychiatr Res	Q1	3.7	10.1016/0022-3956(75)90026-6
4	NIA-AA Research Framework: Toward a biological definition of Alzheimer’s disease	38	Clifford R Jack Jr	2018	Alzheimers Dement	Q1	13.1	10.1016/J.JALZ.2018.02.018
5	2018 Alzheimer’s disease facts and figures	36	Alzheimer’s Association	2018	Alzheimers Dement	Q1	13.1	10.1016/J.JALZ.2018.02.001
6	Automated classification of Alzheimer’s disease and mild cognitive impairment using a single MRI and deep neural networks	34	Silvia Basaia	2018	Neuroimage Clin	Q2	3.4	10.1016/J.NICL.2018.101645
7	A Deep Learning Model to Predict a Diagnosis of Alzheimer Disease by Using 18F-FDG PET of the Brain	34	Yiming Ding	2019	Radiology	Q1	12.1	10.1148/RADIOL.2018180958
8	Deep Residual Learning for Image Recognition	33	Kaiming He	2016	IEEE Conference on Computer Vision and Pattern Recognition (CVPR)			10.1109/CVPR.2016.90
9	The Alzheimer’s Disease Neuroimaging Initiative (ADNI): MRI methods	27	Clifford R Jack Jr	2008	J Magn Reson Imaging	Q1	3.3	10.1002/JMRI.21049
10	Toward defining the preclinical stages of Alzheimer’s disease: recommendations from the National Institute on Aging-Alzheimer’s Association workgroups on diagnostic guidelines for Alzheimer’s disease	27	Reisa A Sperling	2011	Alzheimers Dement	Q1	13.1	10.1016/J.JALZ.2011.03.003

### 3.6 An analysis of the authors and co-cited authors

**Figure 7:**
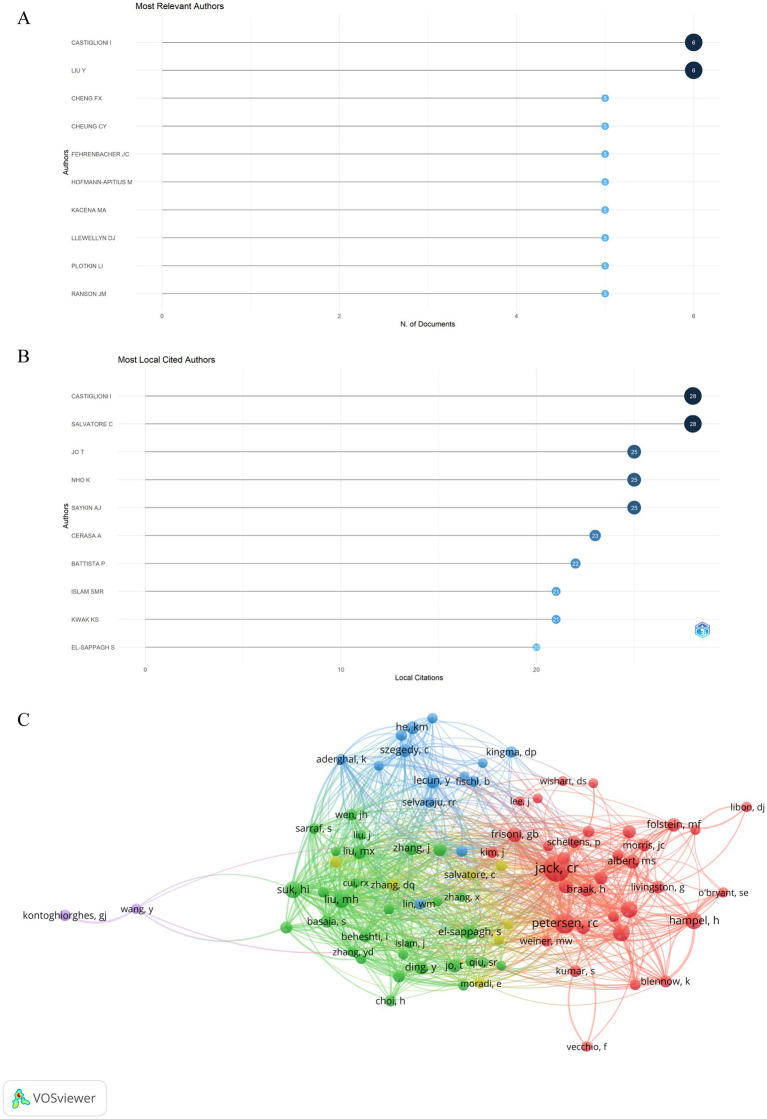
Analysis of active authors engaged in AI-related AD research. **(A)** Top 10 authors in terms of publications. **(B)** Top 10 cited authors in terms of publications. **(C)** The co-citation network map of authors obtained by the VOSviewer. Node size represents the number of citations. The distance between two nodes indicates the relatedness of authors based on the co-citation link. The smaller the distance, the stronger the relatedness, as indicated by similar colored clusters.

A total of 2,900 authors published related articles on artificial intelligence and Alzheimer’s disease based on the visualization chart of 530 screened publications. Figure 7A displays the top 10 authors ranked by publications. Among the authors, Castiglioni I and Liu Y are the leading contributors with the highest number of articles published, each having published 6 articles. Among the most local citation authors (Figure 7B), CASTIGLIONI I and SALVATORE C rank first, with a total of 28 local citations, respectively. Followed by JO T (25 local citations), NHO K (25 local citations) and SAYKIN AJ (25 local citations). We analyzed 24,305 co-cited authors, and those cited over 20 times were visually analyzed, as shown in Figure 7C, there were 87 authors who were cited more than 20 times in the analysis. Among them, 7 authors whose articles were cited more than 50 times, JACK CR stands out with the highest number of co-citations, followed closely by PETERSEN RC and SUK HI, which indicates their research is highly regarded and influential.

### 3.7 An analysis of the keywords

**Figure 8:**
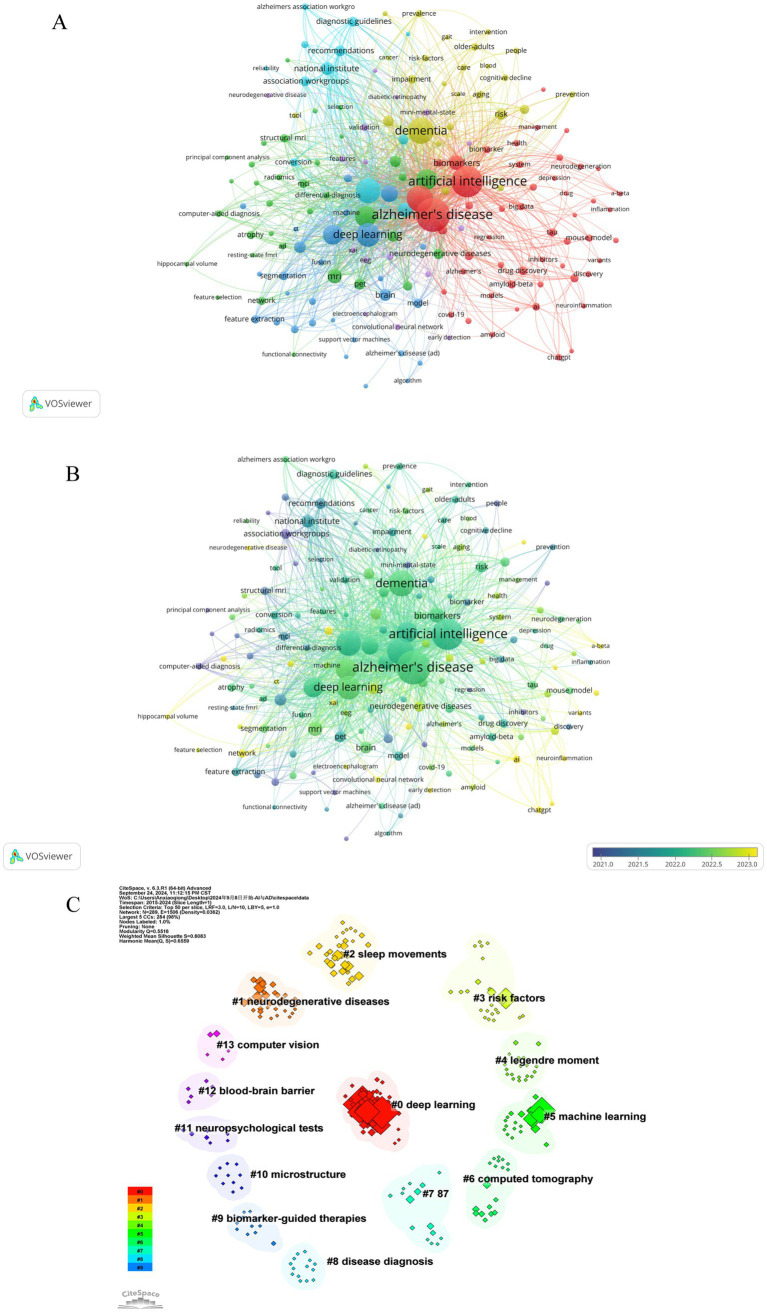
Co-occurrence analysis of keywords. **(A)** Network visualization map of keywords. A keyword is represented by an item, and the size of an item indicates how frequently it appears. Clusters are represented by different colors, and links represent how frequently it appears. **(B)** Overlap visualization map of keywords. The keyword analysis is broken down by average publication year (blue for earlier publications, yellow for later publications). **(C)** Visualization of cluster analysis of keywords.

Keywords, as standardized and specialized vocabulary, provide a comprehensive reflection of the research subject matter. Analyzing frequently occurring keywords visually can facilitate a clearer understanding of the focal points and trends within the area of study. As a result of our analysis, we identified 2,544 keywords related to artificial intelligence and Alzheimer’s disease. Table 5 presents the top 20 most frequently occurring keywords along with their cumulative link strength. In the visualization, only keywords appearing at least 5 times were included, resulting in a total of 162 keywords that met the criteria and were displayed using VOSviewer. A similar colored node indicates that two keywords are within the same cluster, and the size of a node indicates the occurrence frequency of a keyword, whereas the distance between two words indicates the strength of a relationship between two words (Figures 8A,B). The most frequently used keywords were “Alzheimer’s disease,” “artificial intelligence,” “dementia,” “machine learning,” and “mild cognitive impairment” and so on (Table 5; Figures 8A,B).

**Table 5:** The top 20 keywords in artificial intelligence and Alzheimer’s disease research.

Rank	Keyword	Occurrences	Total link strength	Rank	Keyword	Occurrences	Total link strength
1	Alzheimer ‘s disease	152	2,714	11	MRI	45	315
2	Artificial intelligence	100	1,253	12	Disease	45	271
3	Dementia	60	628	13	National institute	36	314
4	Machine learning	50	852	14	Magnetic resonance imaging	29	227
5	Mild cognitive impairment	44	800	15	Brain	29	204
6	Deep learning	41	422	16	Recommendations	25	229
7	Diagnosis	33	510	17	Neuroimaging	25	204
8	Classification	28	392	18	Pet	25	175
9	Prediction	26	543	19	Risk	25	167
10	Biomarkers	24	188	20	Cognitive impairment	24	163

Keywords that were more closely related were grouped into the same cluster and assigned the same color, thereby roughly indicating the research emphasis in recent studies. As show in Figure 8A, based on the results of this study, selected keywords can be roughly divided into 6 clusters, the cluster analysis revealed six distinct groups of keywords, each color-coded to reflect research themes. The red cluster, comprising 49 keywords, was predominantly associated with “Alzheimer's disease”, “artificial intelligence” and “machine learning brain”. The green cluster, containing 43 keywords, featured key terms like “deep learning”, “prediction” and “magnetic resonance imaging”. The blue cluster, with 29 keywords, concentrated on areas such as “dementia”, “biomarkers”, “risk” and “cognitive impairment”. The yellow cluster encompassed 17 keywords, highlighting “early detection”, “automated detection” and “artificial neural network”. The purple cluster, with its 12 main keywords, focused on “mild cognitive impairment”, “national institute” and “diagnostic guidelines”. Lastly, the light blue cluster primarily included “diagnosis”, “neuroimaging” and “PET”. Figure 8B presents an overlay visualization of author keywords, with blue indicating earlier appearances and yellow signifying more recent terms. In the past, keywords like “discovery” “image classification”, “association workgroups” and “computer-aided diagnosis” dominated the discussion, while recent topics include “early detection”, “networking”, “ChatGPT” and “AI”. In addition, CiteSpace was utilized to perform a log-likelihood ratio clustering analysis on the collected keywords, resulting in the identification of 14 distinct clusters, as depicted in Figure 8C. The most prominent clusters are Cluster #0, associated with deep learning, Cluster #1, focusing on neurodegenerative disease, Cluster #2, centered on sleep movements, and Cluster #3, concerning risk factors.

## 4 Discussion

Before 2010, Alzheimer’s treatments primarily managed symptoms without curing the disease. Traditional drugs like acetylcholinesterase inhibitors (donepezil, galantamine, lismin) and the NMDA receptor antagonist memantine aimed to enhance cognitive function and daily living but did not halt disease progression or reverse pathology (31). This study utilizes bibliometric analysis to systematically assess the research landscape surrounding the use of artificial intelligence (AI) in Alzheimer’s disease, concentrating on literature published from 2010 to 2024. By examining publication trends, identifying key contributors, and exploring thematic developments, the research seeks to highlight the role of AI in early diagnosis, disease monitoring, and the formulation of treatment strategies. The results indicate a significant increase in publications over the past few years, demonstrating a growing interest and potential in utilizing AI technologies to tackle the challenges associated with Alzheimer’s disease research (32, 33). Analysis of publication trends reveals a significant increase in research on the applications of artificial intelligence (AI) in Alzheimer’s disease over the past decade, particularly from 2019 to 2024. In this period, 530 publications were recorded, with 361 being original research articles. This indicates a growing interest in the intersection of AI and neurodegenerative disorders. This increase is attributed to advancements in AI technologies that have facilitated new diagnostic and therapeutic approaches in clinical practice. AI’s ability to analyze complex datasets, improve predictive modeling, and enhance patient outcomes has clearly driven further research in this field (32, 34).

Contributions from various countries highlight the collaborative efforts in AI research focused on Alzheimer’s disease. Leading nations like the United States, China, and India show different research priorities and strengths, combining both established and emerging scientific capabilities. The United States leads with 152 publications, underscoring its dominance in biomedical research and innovation. In contrast, China’s rapid growth is evident with 100 publications. This highlights its emerging role as a key player in scientific research, particularly in applying AI to healthcare. Moreover, the analysis shows that international collaborations are crucial. They facilitate knowledge sharing and resource allocation, leading to a more unified strategy for tackling Alzheimer’s disease with AI (35, 36).

An analysis of research output indicates that top institutions are leading the rapidly growing field of Alzheimer’s disease research. For example, Harvard University has published 47 papers. This underscores the significant influence prestigious institutions have on shaping research priorities and outcomes. The robust collaborative network, especially from Harvard Medical School, illustrates how the reputation of institutions can significantly affect both the quality and quantity of research output. However, the relationship between competition and collaboration among these institutions requires further investigation, as it may influence the pace of scientific discoveries and the overall landscape of artificial intelligence research in Alzheimer’s disease. Understanding these institutional dynamics is crucial for fostering an environment that encourages innovation and successfully translates research findings into clinical applications (37, 38).

This investigation reveals that influential literature on AI and Alzheimer’s disease is concentrated in specific journals, particularly the “Journal of Alzheimer’s Disease” and “Alzheimer’s and Dementia.” The high citation rates of these journals show that they are important platforms for sharing critical findings with the scientific community. Citation analysis also shows how these leading journals facilitate knowledge exchange and support the growth of new research areas. This has important implications for researchers. By choosing high-impact journals, they can increase their visibility and connect with a larger audience (39, 40).

An analysis of keywords provides novel insights into the thematic emphases of contemporary research. The prevalence of keywords such as “Alzheimer’s disease,” “artificial intelligence,” and “machine learning” underscores the prominence of these concepts within the scholarly discourse. Furthermore, the organization of keywords into distinct thematic clusters highlights the development of specific research areas, including predictive analytics, imaging techniques, and biomarker identification. This analysis serves as a strategic framework for future research endeavors, delineating prospective pathways for exploration that correspond with contemporary scientific interests and clinical imperatives. Moreover, monitoring the temporal evolution of keywords can yield significant insights into changing research priorities and the assimilation of innovative technologies in Alzheimer’s disease research, with particular emphasis on the integration of deep learning and advanced data analytics (41, 42).

This study has several limitations that should be carefully considered. First, our dependence on the Web of Science database for literature retrieval may have led to the omission of important contributions found in other reputable databases, such as PubMed and CNKI. Furthermore, focusing solely on English-language publications limits our analysis, potentially leaving out valuable insights from research published in other languages. Although we aimed to provide a thorough overview of the research landscape, the lack of standardized criteria for assessing the quality of the literature could introduce biases, especially in how we interpret the findings. To achieve a more comprehensive understanding of the field, future studies should include a wider variety of databases and languages, as well as implement strict quality assessment protocols.

## 5 Conclusion

In conclusion, our systematic analysis of the literature regarding artificial intelligence in the context of Alzheimer’s disease highlights the swift advancement and increasing interest in this interdisciplinary field. By identifying key contributors, influential journals, and emerging research themes, we offer valuable insights that can steer future research and inform clinical practices. As technology and healthcare continue to converge, it is essential for researchers to stay updated on these developments, encouraging collaboration and innovative strategies to improve diagnostic and therapeutic methods for Alzheimer’s disease.

## Data Availability

The original contributions presented in the study are included in the article/supplementary material, further inquiries can be directed to the corresponding authors.

## Footnotes

^1^https://bibliometric.com/
